# Healing Mechanism of Ruptured Fetal Membrane

**DOI:** 10.3389/fphys.2020.00623

**Published:** 2020-06-17

**Authors:** Haruta Mogami, R. Ann Word

**Affiliations:** ^1^Department of Gynecology and Obstetrics, Kyoto University Graduate School of Medicine, Kyoto, Japan; ^2^Department of Obstetrics and Gynecology, Cecil H. and Ida Green Center for Reproductive Biology Sciences, University of Texas Southwestern Medical Center, Dallas, TX, United States

**Keywords:** premature rupture of membrane, fetal membrane, amnion, macrophage, wound healing

## Abstract

Preterm premature rupture of membranes (pPROM) typically leads to spontaneous preterm birth within several days. In a few rare cases, however, amniotic fluid leakage ceases, amniotic fluid volume is restored, and pregnancy continues until term. Amnion, the collagen-rich layer that forms the load-bearing structure of the fetal membrane, has regenerative capacity and has been used clinically to aid in the healing of various wounds including burns, diabetic ulcers, and corneal injuries. In the healing process of ruptured fetal membranes, amnion epithelial cells seem to play a major role with assistance from innate immunity. In a mouse model of sterile pPROM, macrophages are recruited to the injured site. Well-organized and localized inflammatory responses cause epithelial mesenchymal transition of amnion epithelial cells which accelerates cell migration and healing of the amnion. Research on amnion regeneration is expected to provide insight into potential treatment strategies for pPROM.

## Is Pprom Irreversible?

Preterm premature rupture of membranes (pPROM) is a leading cause of preterm birth (Menon and Richardson, 2017). Fetal membrane rupture has traditionally been regarded as an irreversible process: the mean latency period from membrane rupture to delivery is 12 days at 20–26 weeks of gestation and 4 days at 32–34 weeks of gestation (Parry and Strauss, 1998). In some cases, however, ruptured fetal membranes can spontaneously “reseal”: Johnson reported that membrane resealing, defined as cessation of fluid leakage and negative nitrazine test, occurred in 24 cases of 208 pPROM patients (11.5%) in all 5,937 deliveries (Johnson et al., 1990). In addition, we know that the membrane repairs itself and heals spontaneously after amniocentesis (Borgida et al., 2000). These findings suggest that, although most women who experience pPROM deliver spontaneously within several days, the amnion has the capacity for wound healing *in vivo*.

## Causes of Pprom

About 30% of pPROM cases are caused by intra-amniotic infection, whereas the other 70% are unrelated to infection (Romero et al., 1988). pPROM cases that are unrelated to infection are caused by smoking, low body mass index, maternal stress or undernutrition, oxidative stresses, intrauterine bleeding, and iatrogenic factors such as amniocentesis or fetoscopy. Romero et al. reported that intra-amniotic inflammation occurs in 37% of cases of preterm labor before 37 weeks of gestation. Interestingly, the rate of inflammation with infection was only 11%, whereas that of sterile inflammation in the absence of bacteria was 26% (Romero et al., 2014). They suggested that sterile intra-amniotic inflammation might be caused by damage-associated molecular patterns (DAMPs), such as high-mobility group box1 (HMGB1), and concluded that sterile inflammation is a more common contributor to preterm labor than bacterial infection.

DAMPs are believed to play a major role in the pathophysiology of sterile inflammation. Specifically, when a tissue is damaged, intracellular components and molecules such as HMGB1, nucleic acids, heat-shock proteins, adenosine triphosphate, hydrogen peroxide, and calcium ions are released (Kono and Rock, 2008). Uric acid and S100 proteins are associated with pPROM (Friel et al., 2007; Nadeau-Vallee et al., 2016). These DAMPs are recognized by toll-like receptors and receptor for advanced glycation end products (RAGE), leading to activation of inflammatory pathways such as NF-κB and AP-1, which yield sterile inflammation (Akira et al., 2006; Xia et al., 2017). Although DAMPs are released when tissue is damaged, they are also signals of tissue repair. Whereas pPROM initiated by bacterial infection requires immediate delivery to avoid fetal infection, the numerous pPROM cases that are unrelated to infection may be eligible for expectant management.

## Healing of Fetal Tissues: the Roles of Macrophages

The healing mechanisms of adult tissue are divided into four overlapping stages: (1) hemostasis, (2) inflammation, (3) migration and proliferation, and (4) resolution and remodeling (Sonnemann and Bement, 2011). In contrast with adult tissues, the healing of fetal tissue is much simpler (Sonnemann and Bement, 2011): inflammation is suppressed to a minimum, fetal tissue is usually not vascularized, and granulation tissue is usually not formed. These characteristics of fetal wound healing enable the tissue to heal quickly and scarlessly (Cordeiro and Jacinto, 2013). For example, when fetal skin is injured, actin and myosin proteins aggregate in the injured epidermis to form acto-myosin complexes that cause contraction of the tissue and shrinkage of the area of injury. These cellular structures stimulate migration of the epidermis and closure of the wound.

Remarkably, macrophages are recruited to injury sites to facilitate healing of fetal tissues. Circulating monocytes migrate to injury sites where they differentiate into tissue macrophages, and tissue-resident macrophages are also involved in wound healing (Jenkins et al., 2011).

Macrophages are roughly divided into two types (Murray and Wynn, 2011), classically activated macrophages (M1 macrophages) and alternatively activated macrophages (M2 macrophages) (Gordon and Martinez, 2010). Wound healing is facilitated by M2 macrophages (Murray and Wynn, 2011). These cells release growth factors, such as transforming growth factor (TGF-β) and platelet-derived growth factors (PDGF), which activate damaged epidermis and fibroblasts. TGF-β plays a major role in the differentiation of fibroblasts from myofibroblasts. These cells migrate and contract, as well as release tissue inhibitor of metalloproteinases (TIMPs), which inhibits matrix metalloproteinases (MMPs) and prevents over-destruction of tissues. Myofibroblasts also release collagen and repair damaged sites in conjunction with macrophages, which also release MMPs and TIMPs and remodel wounded tissue. Subsequently, macrophages phagocytose debris and damaged extracellular matrix (ECM) to clean the wounded tissues.

## Healing of Amnion in Organ Culture

In an experiment reported by Devlieger et al. (2000b), small holes were generated with a biopsy punch in the centers of human fetal membrane sample. Interestingly, increased cellularity, survival, and proliferation were limited at the tissue border and the rupture did not heal even after 12 days. This result suggests that amnion cannot heal by itself; rather, the help of other cells such as immune cells are necessary for wound healing in the amnion.

## Animal Models of Fetal Membrane Healing

Amnion has a high tensile strength; in fact, the strength of the fetal membrane is provided exclusively by the amnion (Parry and Strauss, 1998). Although fetal membrane structures differ among mammals, humans, and several experimental animals including mice, rats, rabbits, and sheep all have similar amnion structure; they also all have amnion in the most superficial layer of the fetal membrane (Carter, 2016). Thus, animal models are useful for the study of ruptured human fetal membranes *in vivo*.

The first histological observations of the healing process in fetal membranes were conducted in rats. Pioneering work by Sopher (Sopher, 1972) demonstrated that puncturing rat gestational sacs with a 21-gauge needle on day 15 of gestation resulted in a proliferation of amnion mesenchymal cells at the edge of the amnion within 24 h. Further, she showed that the thickened edge of the amnion was covered by epithelial cells and confirmed that wound closure occurred within a few days. Similarly, in a rabbit model, amnion integrity recovered to 40% of its initial value within 30 days of puncture (Deprest et al., 1999). The healing process of rabbit pPROM involves matrix remodeling by MMPs and TIMPs (Devlieger et al., 2000a).

Using a mouse model, we investigated the mechanisms of wound healing of fetal membranes. On day 15 of pregnancy, fetal membranes were mechanically ruptured with sterile needles of various sizes through the myometrium. Ruptured fetal membranes were clearly observed after 6 h and healing began within 24 h. Our mouse study revealed that the closure of such ruptures was complete within 48–72 h (Mogami et al., 2017). Consistent with Sopher’s study, we observed an aggregation of amnion mesenchymal cells at the edge of the amnion at 24 h. Interestingly, this thickened edge was covered by a monolayer of epithelial cells. The proinflammatory cytokines IL-1β and TNF were quickly increased at the fetal membrane rupture site. When a 26-gauge needle was used to create a small rupture, this increase in proinflammatory cytokines returned to basal levels around 24 h. When a 20-gauge needle was used to create a larger rupture, the puncture-induced increases in these cytokines persisted for a longer time. At the same time, IL-10, an anti-inflammatory cytokine, increased at the ruptured site, decelerating inflammation. IL-10 assists in wound healing, as shown by the finding that overexpression of IL-10 in mice accelerates skin healing (Peranteau et al., 2008). In contrast, chronic inflammation conditions such as diabetic ulcers delay wound healing, suggesting the importance of a balance between inflammation and anti-inflammation for complete and organized wound healing. In the amnion, well-controlled switching from a pro‐ to an anti-inflammatory state seems to be necessary for repair.

We observed an aggregation of macrophages around the sterile ruptured amnion (Mogami et al., 2017). These macrophages were fetal-derived and were probably recruited from the amniotic fluid, although they may have been amnion-resident macrophages. These fetal-derived macrophages released IL-1β and TNF at the ruptured site. In contrast with the typical wound healing process in adults, migration of neutrophils was rarely observed. Perhaps this is not surprising given the absence of infection and the sterile nature of the inflammatory stimulus. Yet, this raises questions regarding the role of these inflammatory cytokines at the ruptured amnion. We tested the function of these cytokines through *in vitro* scratch assays using primary human amnion cells. IL-1β and TNF caused significant acceleration of amnion epithelial cell migration. They did not, however, alter amnion mesenchymal cell migration. Importantly, the shape of the amnion epithelial cells changed, assuming a more spindle-like configuration (similar to that of mesenchymal cells) at the edge of migration. These spindle-shaped cells were immunoreactive for vimentin, suggesting that these wounded epithelial cells were undergoing epithelial-mesenchymal transition (EMT). *In vivo*, similarly, vimentin-positive cells can be observed scattered in the epithelial layer of the ruptured amnion in mice, suggesting that EMT occurs *in vivo* as well. EMT is known to speed up cell migration, which in turn speeds up wound closure. Our results imply that EMT provides more mesenchymal cells to the wounded amnion, where these cells then synthesize and release extracellular matrices such as collagen to strengthen the injured site. Richardson and Menon also reported that EMT occurs during amnion healing (Richardson and Menon, 2018) and that mesenchymal-epithelial transition (MET) occurs with the help of IL-8 once amnion closure is complete. In addition, Richardson et al. also recently showed that oxidative stresses activate the p38 MAPK pathway, which causes EMT in the fetal membrane (Richardson et al., 2020). Taken together, these results suggest that EMT is a key mechanism involved in stimulating amnion healing in the presence of sterile inflammation.

There is a concern that the healing properties of the amnion differ among species. In rabbits, for example, relatively small punctures created with a 14-gauge needle spontaneously healed to 41.7% of their initial state (Deprest et al., 1999), whereas relatively large ruptures created with a 1 cm hysterotomy did not heal at all (Papadopulos et al., 1998). Similarly, in a mouse model, the amnion healed at a slower rate after being punctured with a 20-gauge needle than after being punctured with a 26-gauge needle (Mogami et al., 2017). We speculate that the reported variation in healing potential depends on the initial size of the rupture rather than on species differences.

## Importance of “Scaffolds” For Healing Tissues

ECM scaffolds have recently received attention as a fascinating mechanism involved in wound healing acceleration and tissue regeneration (Eming et al., 2014). For example, a type-1 collagen patch preserved contractility and protected cardiac tissue from injury in a mouse myocardial infarction model, accompanied by attenuated left ventricular remodeling, diminished fibrosis, and formation of a network of blood vessels within the infarct (Serpooshan et al., 2013; Wei et al., 2015). Porcine urinary bladder ECM scaffold implantation improved the regeneration of muscle in volumetric muscle loss in rodents as well as in five human patients; perivascular stem cell mobilization was seen in connection with this procedure (Sicari et al., 2014). Bioengineered biomaterials have been clinically applied to replace and restore the skin, heart valves, trachea, and tendons (Lutolf and Hubbell, 2005; Berthiaume et al., 2011).

The application of biomaterials to ruptured membranes has been attempted in such animal models as rabbits, sheep, and rats (Zisch and Zimmermann, 2008). When gelatin sponge plugs were used in ewes and rhesus monkeys, for example, rupture sites were found to be intact at term (Luks et al., 1999).

**Figure 1:**
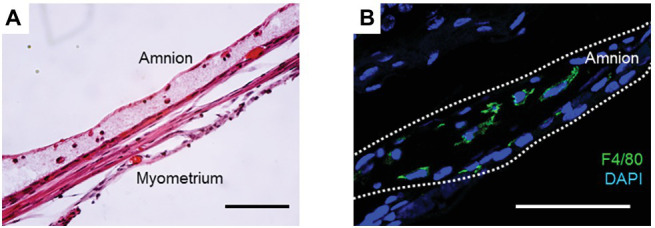
**(A)** H&E staining of collagen-injected fetal membrane at ruptured site at 72 h. Note that a collagen gel layer was formed beneath the amnion, and immune cells were trapped inside the gel. **(B)** Immunofluorescence staining for F4/80 (green) and DAPI (blue) in the collagen layer at 48 h. Bars, 50 μm. All animals were handled and euthanized in accordance with the standards of humane animal care described by the National Institutes of Health Guide for the Care and Use of Laboratory Animals, using protocols approved by the Institutional Animal Care and Use Committee (IACUC) of the University of Texas Southwestern Medical Center.

Previously, we showed that application of a collagen matrix assisted amnion healing in a mouse model of sterile pPROM (Mogami et al., 2018). In this model, a type I collagen gel was injected into mechanically-ruptured sites on murine fetal membranes immediately after puncture. The collagen gel was immediately solidified due to the animal’s body temperature such that it formed a collagen matrix layer beneath the ruptured amnion (Figure 1A). Interestingly, macrophages were trapped in this layer of collagen (Figure 1B). Moreover, this injection of collagen thickened the healing site, presumably stimulating more collagen synthesis by the mesenchymal cells in the amnion. We found vimentin-positive mesenchymal cells in the wounded layer of the amnion, suggesting that EMT occurs in this situation, as we had previously reported in our mouse pPROM model. Collagen injection dramatically increased the overall healing rate to 90%, whereas an injection of phosphate buffered saline alone resulted in a healing rate of only 40%. We concluded that scaffold formation at the wounded site in the amnion stimulates wound healing through at least two mechanisms. First, the scaffold provides a base for migrating amnion cells to cover the wound. Second, the matrix scaffold traps, concentrates, and localizes wound healing macrophages.

Application of collagen to the rupture site has also been tested in a rabbit pPROM model. In that study, amnion integrity was diminished by the injection of a collagen “plug” compared to myometrial closure alone. This result is different from ours. We speculate that this is because we injected a collagen “gel” in liquid form to the rupture site using a syringe, such that the gel spreads immediately after injection around the rupture site rather than forming a “plug” as in the rabbit study (Papadopulos et al., 1998). The formation of a plug might block the migration of amnion cells. Our collagen gel, in contrast, formed a collagen layer beneath the amnion in our mouse model. This layer serves as a scaffold for migrating amnion cells and traps macrophages. Thus, it never interferes with the healing process. The form of biomaterials (liquid or solid) and the means of their application (injection or patch) may thus be as important as the material type itself.

The effectiveness of biomaterial scaffolds has been observed in other tissues. Bone and cardiac muscle-derived tissue ECM scaffolds for traumatic muscle wounds in mice improved tissue regeneration (Sadtler et al., 2016). In this study, macrophages and immune cells were increased at the injured site, allowing these immune cells to be polarized into a type 2 immune state. Therefore, providing a scaffold is a good strategy for stimulating healing of ruptured amnion. The least invasive means of accomplishing this *in vivo* remains under active investigation.

## Conclusion

Based on several previous studies, we speculate that the amnion might be capable of healing. Several cell types coordinate and orchestrate wound healing in the fetal membranes, including amnion epithelial cells that differentiate into mesenchymal cells, migrating mesenchymal cells, differentiating resident macrophages, and recruited fetal macrophages. ECM scaffolds could support spontaneous healing of the amnion not only by promoting the migration of amnion cells but also by polarizing macrophages into a type-2 phenotype. The mechanisms by which the amnion heals itself represent a new field of study in which a great deal more research must be done to clarify how this healing process works.

## Author Contributions

HM and RW wrote the manuscript.

## Conflict of Interest

The authors declare that the research was conducted in the absence of any commercial or financial relationships that could be construed as a potential conflict of interest.
